# Hodgkins disease

**DOI:** 10.1054/bjoc.2001.1765

**Published:** 2001-05

**Authors:** C Ferme, J M Cosset, B Fervers, C Sebban, B Cutuli, M Henry-Amar, J Stines, F Giammarile, P Bey, T Philip

**Affiliations:** Hôpital Saint-Louis, Paris; Institut Curie, Paris; Centre Léon Bérard, Lyon; Cancer Research Campaign, Paris; Centre Paul Strauss, Strasbourg; Centre Francois-Baclesse, Caen; Centre Alexis Vautrin, Nancy; Hôpital San Martino, Gènes, Italie


British Journal of Cancer (2001) 84 (Supplement 2), 55–60
© 2001 FNCLCC
doi: 10.1054/ bjoc.2001.1765, available online at http://www.idealibrary.com on IDEAL®

### Hodgkins disease

C Ferme[1], JM Cosset[2], B Fervers[3,4], C Sebban[3], B Cutuli[5], M Henry-Amar[6], J Stines[7], F Giammarile[3], P Bey[7], AM Carella[8] and T Philip[3]

[1]Hôpital Saint-Louis, Paris; [2]Institut Curie, Paris; [3]Centre Léon Bérard, Lyon; [4]FNCLCC, Paris; [5]Centre Paul Strauss, Strasbourg; [6]Centre Francois-Baclesse, Caen; [7]Centre Alexis Vautrin, Nancy; [8]Hôpital San Martino, Gènes, Italie

The development of extended-field radiotherapy, of chemotherapy and then of the combination of the two therapeutic modalities, has led to cure rates of almost 80% in patients with Hodgkins disease (HD). The aim of management in patients presenting with HD must no longer be cure alone, but cure without sequelae and with the best possible quality of life. Further efforts are necessary to reduce the long-term toxicity of treatment and to improve the prognosis of recurrent disease and that resistant to standard treatment. Numerous questions remain unanswered regarding the heterogeneous histology and phenotypes of the disease and the diversity of the clinical presentations.

This document does not apply to patients who develop HD as a consequence of HIV disease. Similarly, the management of patients with nodular lymphocyte-predominant HD (paragranuloma of Poppema), which is considered to be a different entity than HD, is not considered.

The SOR document on the management of HD in adults was published in full in 1998. An update is planned for the year 2001. It will deal in particular with:

- the best combinations of radiochemotherapy and the move away from radiotherapy as single-modality treatment for stage I–II supradiaphragmatic disease
- the role of international prognostic scores in the development of treatment strategies for disseminated disease
- the indications and the best modalities for irradiation in the treatment of advanced disease.

### DIAGNOSIS

The diagnosis of HD depends on the histological examination of a biopsy and the identification of Reed–Sternberg cells (standard). A cytological diagnosis alone is not acceptable. The use of immune markers is necessary for the differential diagnosis in lymphocyte-predominant types (CD30, CD15, CD45, EMA, CD20). It is recommended for other forms, such as lymphocyte-depleted types (CD30, CD45, EMA, T and B markers) of the Lukes–Rye classification (option), in order not to misdiagnose anaplastic large cell lymphoma. In difficult cases, review by a specialist pathologist is recommended.

### STAGING

Accurate initial staging is essential in order to classify patients into groups according to specific prognostic factors that define the treatment strategy.

The clinical history provides information about systemic symptoms (fever, sweats and weight loss) that are essential for the classification of the disease (standard). Clinical examination must detect any site of superficial lymphadenopathy, as well as any hepato- or splenomegaly (standard).

Assessment of the chest should include a chest X-ray (AP and lateral) and a thoracic CT scan (standard). A large mediastinal mass is defined as that having a diameter equal or greater than a third of the transverse diameter of the chest at the level of the T5–T6 disc interspace, from a thoracic film taken AP with the patient standing (standard). The place of MRI in the staging of disease in the chest remains to be determined and it must only be used within a trial setting. Routine gallium scans are not indicated during initial staging (level of evidence C). A baseline image of a large mediastinal mass using SPECT (single photon emission computed tomography) can be used to evaluate subsequent response to treatment.

Examination of Waldeyer's ring by a specialist is indicated in cases where there is high cervical lymphadenopathy (standard).

An abdomino-pelvic CT scan is used to detect sub-diaphragmatic disease (standard). A bipedal lymphangiogram and an abdominal ultrasound (option) can provide additional information to that gained from CT scanning. There is no consensus as to the routine use of bipedal lymphography in diagnostic staging (level of evidence C). No study has evaluated the impact of the addition of lymphangiography to CT scanning on treatment decisions. A lymphangiogram is recommended for localized disease where the result would have an impact on treatment planning, in particular when CT scanning has not shown any involvement of sub-diaphragmatic nodes. The interpretation of lymphangiograms requires specialist experience. If imaging has demonstrated focal liver lesions of uncertain aetiology, a guided-needle biopsy under CT or ultrasound control is indicated (option). Ultrasonography is less accurate than CT scanning.

The place of MRI in abdominal staging remains to be determined. Any evaluation of the sensitivity and specificity of MRI and its impact on treatment planning must also consider cost. Gallium scanning has only a limited role in the evaluation of sub-diaphragmatic disease (level of evidence B).

A bone marrow biopsy should be undertaken routinely in advanced-stage disease and limited-stage disease with unfavourable prognostic factors (standard). A bone marrow biopsy is optional in limited-stage disease with favourable prognostic factors. In advanced-stage disease, with involvement above and below the diaphragm and/or the presence of systemic symptoms (B symptoms), bone involvement can be investigated with scintigraphy (gallium or technetium bone scan) or by MRI (level of evidence C). The place of MRI in the investigation of bone marrow involvement remains to be evaluated.

The value of any blood test will depend on its prognostic value and whether or not it will alter treatment decisions. Routine blood tests should include a full blood count and platelets, ESR, liver function and serology for HIV (standard). Lactic dehydrogenase (LDH) or B2-microglobulin levels and protein electrophoresis (option) can be undertaken in advanced-stage disease or within a therapeutic trial.

Initial staging is completed by other baseline investigations necessary prior to treatment with chemotherapy or radiotherapy. Sperm cryopreservation must be considered routinely when chemotherapy or infradiaphragmatic radiotherapy is to be given (standard).

There is no longer any indication for an exploratory laparotomy with splenectomy (standard) (level of evidence B). It can be considered in certain circumstances when the information gained would change the treatment plan, notably if there is doubt regarding the presence of sub-diaphragmatic disease (level of evidence C).

### CLASSIFICATION AND THERAPEUTIC GROUPING

Staging is based on the 'Cotswold' modifications of the Ann Arbor classification (standard). HD can be classified as limited (stage I and II, above or below the diaphragm) or extensive (stage III or IV disease) (standard).

Patients are categorized into therapeutic groups according to both stage and prognostic factors (standard). Limited-stage disease above the diaphragm can be stratified into 'favourable' and 'unfavourable' groups according to the following prognostic factors: age, number of nodal sites involved, ESR, B symptoms and the existence or absence of a large nodal mass (level of evidence B).

Extensive-stage disease can be differentiated into stages IIIA, IIIB and IV (standard). The relevant prognostic factors for stage IIIA disease are age, ESR, the extent of abdominal involvement, splenic disease and the number of sites involved (level of evidence C). The prognostic factors used to plan treatment for stages IIIB and IV disease in therapeutic trials (as recommended by the FNCLCC, the French Adult Lymphoma Study Group (GELA) and the French Bone Marrow Transplantation Group) are: anaemia, level of serum lactate dehydrogenase, inguinal node involvement, large-volume mediastinal disease (defined as a tumour mass ratio greater than 0.45), bone marrow disease and multiple organ involvement.

### TREATMENT MODALITIES

### Surgery

Surgery no longer has a therapeutic role in the management of HD (standard). Surgical biopsy is essential (standard). Exploratory laparotomy with splenectomy is no longer routine practice (standard); for limited-stage disease, it carries no benefit in terms of long-term survival (level of evidence B). The decision to undertake aggressive treatment for patients presenting with bad prognosis disease is independent of the results of a laparotomy/splenectomy (level of evidence C).

### Radiotherapy

Radiotherapy planning must be adapted to the patient and the volume to be treated (standard). For classic 'extended volume' fields (mantle, inverted Y, para-aortic plus spleen) photon beam energy of 10–25 Mv from a linear accelerator is recommended for stage I and II supradiaphragmatic disease. The use of Cobalt is not recommended for mantle irradiation or inverted Y fields in adult patients. Supra-diaphragmatic mantle irradiation includes the bilateral cervical and supraclavicular areas, bilateral axillae, the medias and pulmonary hila (standard). Subdiaphragmatic inverted Y irradiation includes the para-aortic, bilateral external iliac and the bilateral inguinal areas (standard). Irradiation of the para-aortic lymph nodes and spleen (option) is indicated when the iliac and inguinal regions are not to be irradiated, thus sparing the ovaries and the bone marrow (standard).

Treatment volumes can be reduced or increased in certain clinical circumstances (option). After chemotherapy, radiotherapy fields can often be reduced to cover only those areas initially involved (level of evidence C). The doses delivered are those of the ICRU 50 report (standard). If radiotherapy is given as sole treatment, the appropriate dose is 40 Gy to involved areas and 30–36 Gy for 'prophylactic' treatment (level of evidence C). After chemotherapy, the dose to areas initially involved is 36 Gy after complete remission and 40 Gy following incomplete remission (level of evidence C).

When there has been no initial mediastinal involvement, the dose to the inferior mediastinum for subtotal nodal irradiation following chemotherapy is reduced to 30 Gy (level of evidence C). It is not recommended to lower the total dose of irradiation except within a therapeutic trial evaluating dose reduction (level of evidence C).

The standard dose rate is 9–10 Gy per week, in five fractions (standard). The recommended dose per fraction is 1.8–2 Gy (level of evidence B). The dose per fraction must not exceed 2 Gy (standard, level of evidence B).

Large fields are irradiated in two planes treated in the same session; antero-posterior and posterior-anterior. If at all possible, dosimetry must ensure maximum homogeneity within the treated volume to avoid heterogeneous treatment which could result in an overdose to certain areas with doses per fraction above 2 Gy (standard). Quality control is mandatory.

Control films should be taken once a week. The minimum requirement is two films during the course of treatment (standard). Custom shielding blocks must be individualized and focused on the relevant area (standard). The patient is treated in the dorsal decubitus position (standard). The ventral decubitus position is not recommended.

### Chemotherapy

Several chemotherapy protocols have proven efficacy: MOPP/ABV (nitrogen mustard, vincristine, procarbazine, prednisone/doxorubicin, bleomycin, vinblastine), MOPP/ABVD (nitrogen mustard, vincristine, procarbazine, prednisone/doxorubicin, bleomycin, vinblastin, dacarbazine), ABVD (doxorubicin, bleomycin, vinblastin, dacarbazine), CVPP (cyclophosphamide, vinblastin, procarbazine, prednisone), BCVPP (carmustine, cyclophosphamide, vinblastin, procarbazine, prednisone) and LOPP (chorambucil, vincristine, procarbazine, prednisone) (options).

The results obtained with ABVD are comparable or superior to those obtained with MOPP, with less long-term toxicity (secondary leukaemia, sterility) (level of evidence B). The protocols containing seven or eight drugs are superior to MOPP (level

© 2001 FNCLCC

of evidence B). The use of MOPP alone is not recommended (level of evidence B).

The ABVD protocol is considered by a number of groups to be the reference chemotherapy. It is recommended that centres become familiar with the use of one protocol (level of evidence B).

### Late toxicity

The two most important complications which can cause late deaths in patients cured after treatment of HD are cardiac (myocardial infarction) and second cancers (leukaemia and solid tumours). The onset of hypothyroidism may be very late. It is important to inform patients before treatment about the potential acute, median-term and long-term toxicities (standard).

Limiting the dose to the lower mediastinum to 30 Gy, or excluding this area completely from the irradiation field, is recommended if at all possible in order to reduce the risk of late myocardial infarction (level of evidence C).

Ovariopexy should be undertaken routinely before iliac irradiation in young women (standard). The inclusion of patients in therapeutic trials evaluating the possibility of reducing therapy in order to reduce late toxicity is recommended (level of evidence B).

### THERAPEUTIC STRATEGY

### Stage I and II supra-diaphragmatic disease 'favourable' group

Current studies are evaluating sub-total nodal irradiation and chemoradiotherapy combinations for the treatment of this group (Figure 1, left arm). While survival rates are equivalent (level of evidence B), the incidence of long-term complications might favour protocols with a reduced number of cycles of chemotherapy and limited radiotherapy as compared to irradiation alone (level of evidence C). The indication for chemotherapy alone remains controversial (level of evidence C).

It is recommended that patients are included in trials evaluating less toxic protocols, with a reduction in both the number of courses of chemotherapy and/or the dose of radiotherapy (level of evidence C).

### Stage I and II supra-diaphragmatic disease 'unfavourable' group

The standard treatment is combination chemoradiotherapy (Figure 1, right arm). Irradiation alone is not recommended (level of evidence C). The recommended treatment is six courses of chemotherapy followed by irradiation to initially involved sites (level of evidence B). The inclusion of patients in therapeutic trials evaluating the optimal combination of treatment modalities is recommended.

### Stage I and II sub-diaphragmatic disease

The frequency of bad prognostic factors in patients with stage I and II disease below the diaphragm (as defined for the unfavourable group of stages I and II supra-diaphragmatic disease) justifies the use of combination chemo- and radiotherapy as standard treatment (i.e. six courses of chemotherapy followed by irradiation to original disease sites) (level of evidence C) (Figure 2). Laparotomy with splenectomy does not alter the therapeutic strategy (level of evidence C), but may be of value in certain specific cases. For clinical stage IA inguino-femoral disease without bad prognostic features, irradiation alone (inverted Y plus spleen) can be considered (option, level of evidence C).

### Stage IIIA disease

Prognostic factors such as the extent of abdominal nodes, the volume of involved nodes (defined according to the Cotswold classification), the age and the ESR, determine treatment for stage IIIA disease (level or evidence B) (Figures 3 and 4). For patients with stage IIIA with no unfavourable features (stage IIIA, age less than 50 years, ESR less than 50 mm $h^{-1}$ and the absence of large volume disease), three therapeutic protocols can be considered (options):

- subtotal nodal irradiation (level of evidence C)
- combination chemoradiotherapy with 3–4 courses of chemotherapy followed by irradiation to sites of initial disease (level of evidence B)
- chemotherapy alone (six courses) provided there is an early complete remission.

For patients with stage IIIA disease with unfavourable features, standard treatment is combination chemoradiotherapy with 4–6 courses of chemotherapy followed by radiotherapy to initial disease sites (level of evidence C). Radiotherapy alone in these patients is not recommended (level of evidence B). The reduction of toxicity should be a primary end-point in trials evaluating novel combination treatment modalities.

### Stage IIIB and IV disease

Standard treatment is 6–8 courses of combination chemotherapy (level of evidence B) (Figures 3 and 5). Radiotherapy has not been shown to confer any extra benefit over chemotherapy alone (level of evidence C). There is insufficient evidence to define the place of intensification therapy for patients with stage IV disease. The inclusion of patients in randomized trials is recommended. Subsequent treatment of stages IIIB and IV disease is determined according to the response to chemotherapy. If induction chemotherapy results in a complete remission, the options are no additional radiotherapy and radiotherapy to original sites of large-volume disease (level of evidence C).

If 6–8 courses of induction chemotherapy have only resulted in a partial remission (greater than 50% response) and in the absence of progressive visceral involvement, irradiation limited to the residual masses is optional (level of evidence C).

The inclusion of these patients in therapeutic trials is recommended, to clarify the role of intensification therapy.

If the response after 6–8 courses of induction chemotherapy is less than 50% or if there is progressive disease, standard treatment consists of 'salvage' treatment with non-cross resistant chemotherapy, with or without intensification therapy using haematopoietic stem cell rescue and radiotherapy (level of evidence C). The inclusion of patients in therapeutic trials is recommended.

### Recurrent disease

There is insufficient evidence to define clearly the best treatment for recurrent HD. In the sub-group of bad-prognosis patients

© 2001 FNCLCC

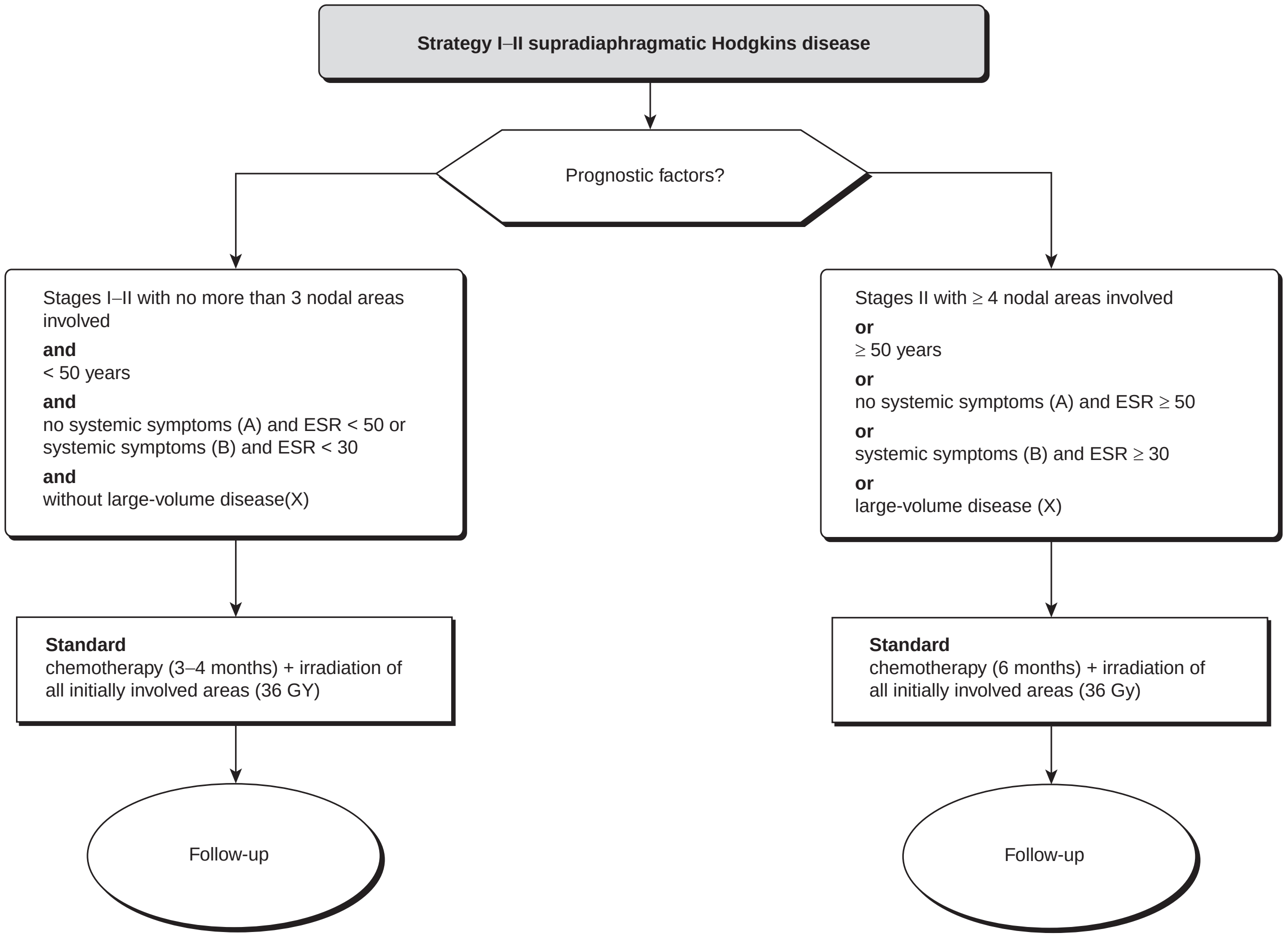

**Figure 1** Treatment of clinical stage I–II supradiaphragmatic Hodgkins disease

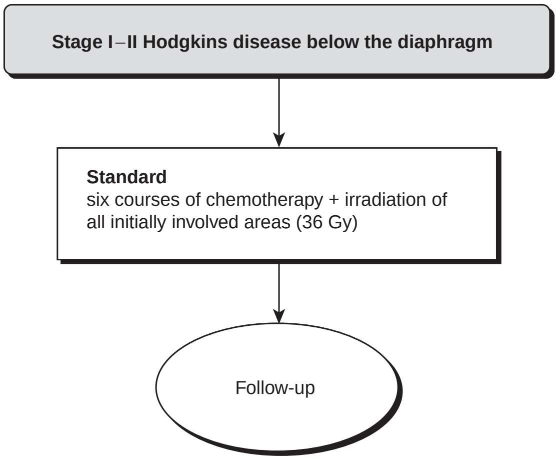

**Figure 2** Treatment of stage I–II disease below the diaphragm

(those with resistant disease or early recurrence), intensive chemotherapy is at least equivalent, if not better, than conventional chemotherapy (level of evidence C). The use of peripheral stem cells and growth factors has reduced the toxicity of intensification (level of evidence B). The treatment of recurrent disease must take into consideration the initial treatment, the duration of complete remission, the stage at recurrence and the age of the patient (level of evidence C).

The use of conventional chemotherapy (with or without radiotherapy) can be considered for the following patients:

- those initially treated with only radiotherapy or MOPP chemotherapy (level of evidence C)
- those with a localized recurrence who have been in complete remission for more than 12 months (level of evidence C)
- those greater than 65 years in age.

In the other cases, an intensification regimen with haematopoietic stem cell rescue is recommended (level of evidence C). This should be undertaken after salvage with second-line chemotherapy. No one conditioning regimen has proven superiority over any other (level of evidence C). It is recommended that any non-irradiated initial disease site is irradiated following the stem cell graft. Patients should be entered into therapeutic trials to clarify the indications for intensification therapy.

### FOLLOW-UP

Follow-up during treatment is based on clinical examination, chest X-ray (CXR) and CT scan (standard). Gallium scanning is recommended for the evaluation and surveillance of residual mediastinal masses (level of evidence B). The place of PET

 © 2001 FNCLCC

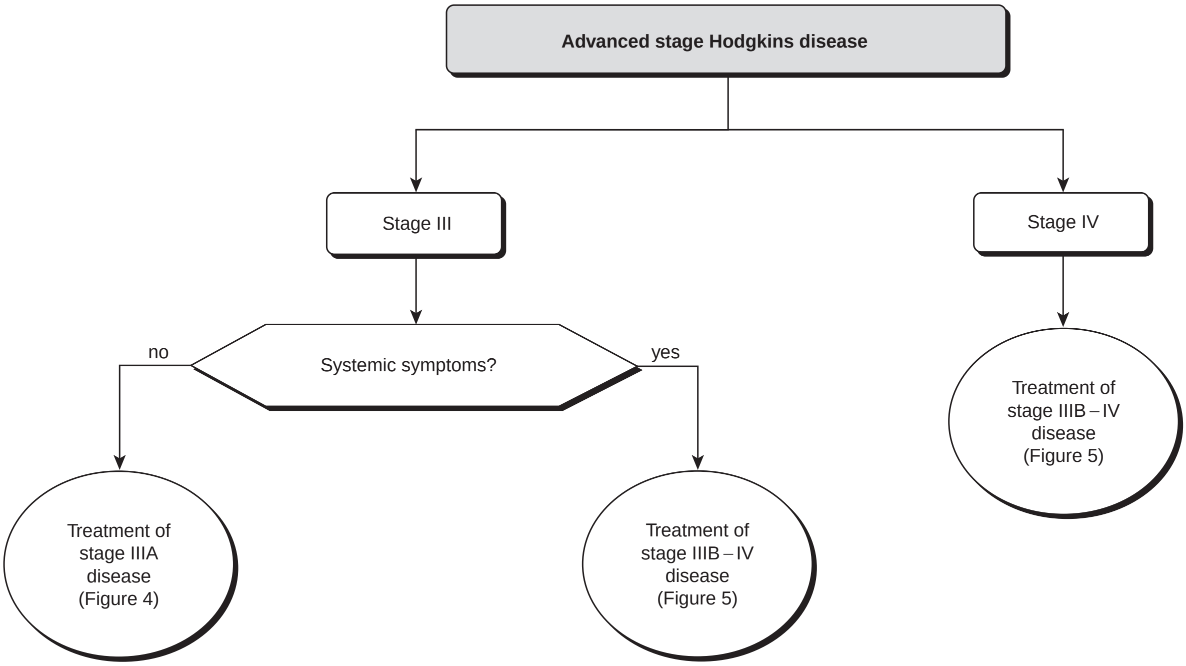

**Figure 3** Treatment of advanced stage Hodgkins disease

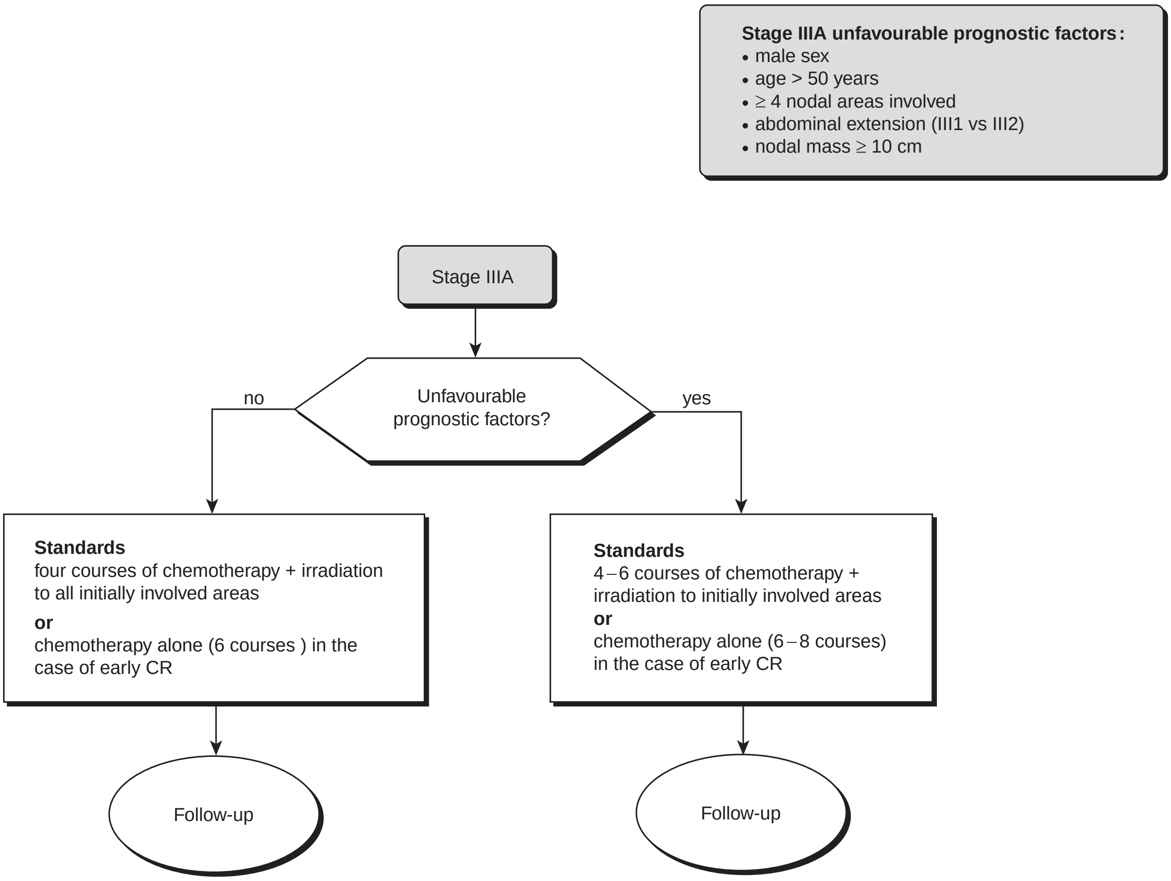

**Figure 4** Treatment of stage IIIA disease

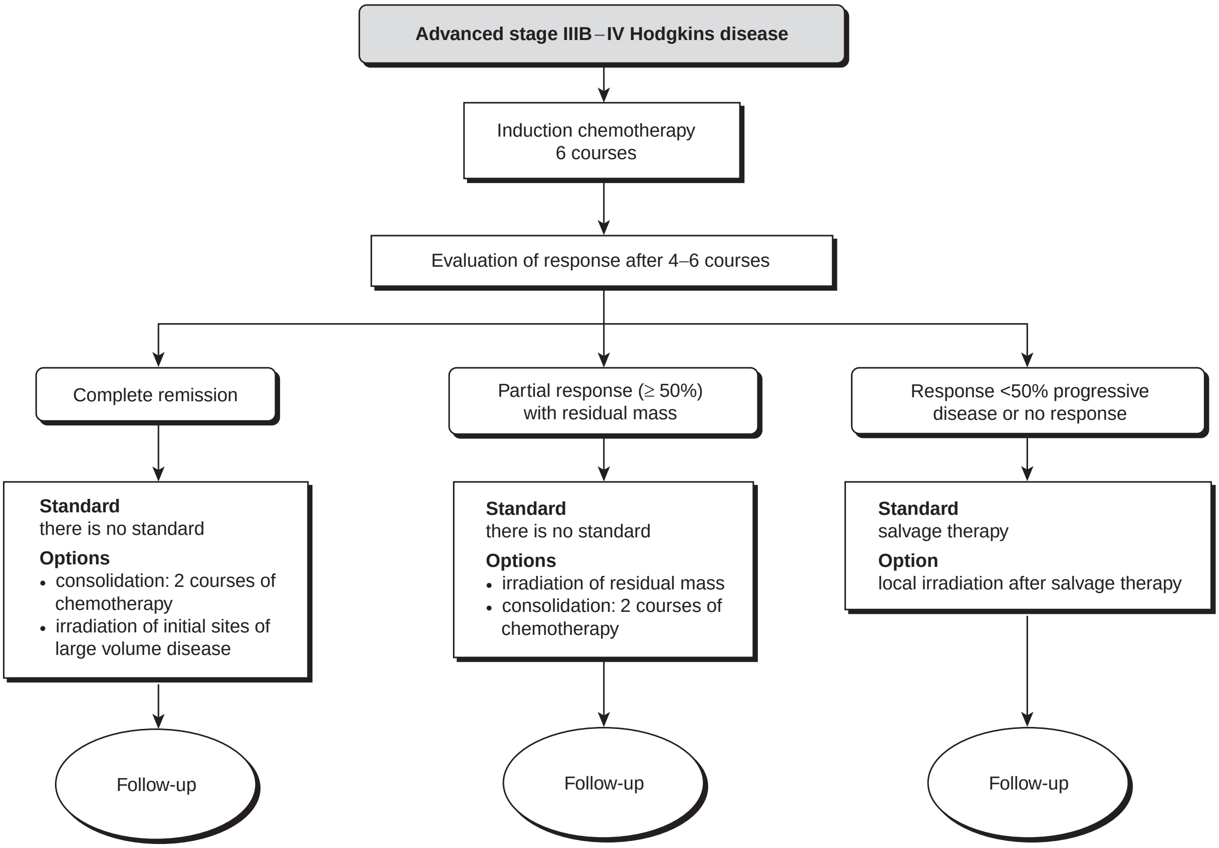

**Figure 5** Treatment of stages IIIB and IV disease scanning (using fluorodesoxyglucose (FDG)) in the assessment of response to therapy is under evaluation. MRI is not recommended in routine practice. In the absence of complications, clinical surveillance can be monthly during treatment. The frequency of chest X-rays and CT scans is adapted to treatment and clinical indication.

The best methods and frequency of follow-up, or the indications for investigations, have not been evaluated. The frequency recommended by the Costwolds Meeting is one consultation every 3 months during the first and second years, every 4 months during the third year and every 6 months during the fourth and fifth years, then each year. This pattern has been adapted for the follow-up of patients after treatment in clinical trials. It consists of a clinical examination, full blood count including platelet count, ESR and a CXR in the case of initial mediastinal involvement. CT scanning is used for the follow-up of patients having focal residual disease above or below the diaphragm, clinical suspicion or blood test abnormalities (option). Late sequelae after treatment (cardiac, respiratory, endocrine, etc) should be anticipated and prospectively evaluated (standard).

### INTERNAL REVIEWERS

I Brenot-Rossi (Institut Paoli Calmettes, Marseille), C Carrie (Centre régional Léon Bérard, Lyon), C Chevreau (Centre Claudius Régaud, Toulouse), C Cohen-Solal Le Nir (Centre René Huguenin, Saint-Cloud), C Couanet (Institut Gustave Roussy, Villejuif), F Dhermain (Centre Henri Becquerel, Rouen), H Eghbali (Institut Bergonié, Bordeaux), M Fabbro (Centre Val d'Aurelle, Montpellier), M Hayat (Institut Gustave Roussy, Villejuif), S Hoffstetter (Centre Alexis Vautrin, Vandœuvre-Lès-Nancy), M Janvier (Centre René Huguenin, Saint-Cloud), D Lebrun (Institut Jean Godinot, Reims), B Le Mevel (Centre René Gauducheau, Nantes), M Monconduit (Centre Henri Becquerel, Rouen), E Netter (Centre Alexis Vautrin, Vandœuvre-Lès-Nancy), TD Nguyen (Institut Jean Godinot, Reims), H Pacquement (Institut Curie, Paris), X Panis (Institut Jean Godinot, Reims), P Soubeyran (Institut Bergonié, Bordeaux), F Soum (Centre Claudius Regaud, Toulouse) and A Tanguy (Centre François Baclesse, Caen).

### EXTERNAL REVIEWERS

F Berger (Hôpital Edouard Herriot, Lyon), B Coiffier (Centre Hospitalier Lyon Sud, Pierre Benite), P Colombat (CHU Hôpital Bretonneau, Tours), A Delmer (Hôtel-Dieu, Paris), G Delsol (CHRU Hôpital Purpan, Toulouse), C Gisselbrecht (Hôpital Saint-Louis, Paris), F Guilhot (CHU Hôpital Jean Bernard, Poitiers), JP Marie (Hôtel-Dieu, Paris), P Romestaing (Centre Hospitalier Lyon Sud, Pierre-Bénite), B Roullet (Hôpital Dupuytren, Limoges), JJ Sotto (CHU Hôpital Nord, Grenoble), B Varet (Hôpital Necker, Paris) and G Ganem (Centre Jean Bernard, Le Mans).

 © 2001 FNCLCC

